# Increased central serotonergic activity in patients after an acute ischemic stroke. An EEG study

**DOI:** 10.1016/j.cnp.2025.10.003

**Published:** 2025-10-17

**Authors:** Vera Flasbeck, Andreas Ebert, Bettina Klostermann, Daniel Richter, Ralf Gold, Christos Krogias, Georg Juckel

**Affiliations:** a Department of Psychiatry, Psychotherapy and Preventive Medicine, LWL University Hospital, Ruhr-University Bochum, Bochum, Germany; b Department of Neurology, St. Josef Hospital, Ruhr University Bochum, Bochum, Germany; c Department of Neurology, EvK Herne, Herne, Germany

**Keywords:** Serotonergic neurotransmission, Loudness dependence of auditory evoked potentials, Acute ischemic stroke, Cerebrovascular event, Post-stroke depression

## Abstract

**Objective:**

Acute ischemic stroke (AIS) is often accompanied by functional impairments and post-stroke depression (PSD), affecting ∼30 % of patients. Since central serotonergic dysfunction may contribute to PSD, similar to major depressive disorder, we examined depressive symptoms and serotonergic neurotransmission after AIS using the loudness dependence of auditory evoked potentials (LDAEP), a non-invasive EEG marker.

**Methods:**

19 patients with AIS and 18 age-matched healthy participants completed depression questionnaires (BDI, HAMD). LDAEP was assessed within 14 days after AIS and after three months, analyzed with mixed-models ANOVA.

**Results:**

Shortly after AIS, patients showed lower LDAEP compared to controls for all electrodes (AIS *M* = 0.072 ± 0.077; controls *M* = 0.133 ± 0.095; *p* = 0.037), frontal (*p* = 0.011) and frontocentral (*p* = 0.027) electrodes. After three months, differences reached trend-level (*p* = 0.12). Depression scores were higher in patients, but not clinically relevant.

**Conclusions:**

AIS appears to be associated with altered serotonergic neurotransmission, with attenuated LDAEP differences at three months possibly reflecting remission. As depression scores were not clinically relevant, serotonergic changes likely reflect a general AIS effect rather than being specific to PSD.

**Significance:**

Early LDAEP measurement may serve as a non-invasive biomarker to identify patients with altered serotonergic functioning and guide interventions, such as SSRI therapy, to reduce PSD risk and improve recovery.

## Highlights

• 14 days after AIS: patients show lower LDAEP than healthy controls, which might indicate increased serotonergic activity.
• At 3 months: patient-control LDAEP gap reached trend level, suggesting recovery.
• AIS could have led to short-term alterations in serotonergic neurotransmission.

## Graphical abstract

**Figure:**
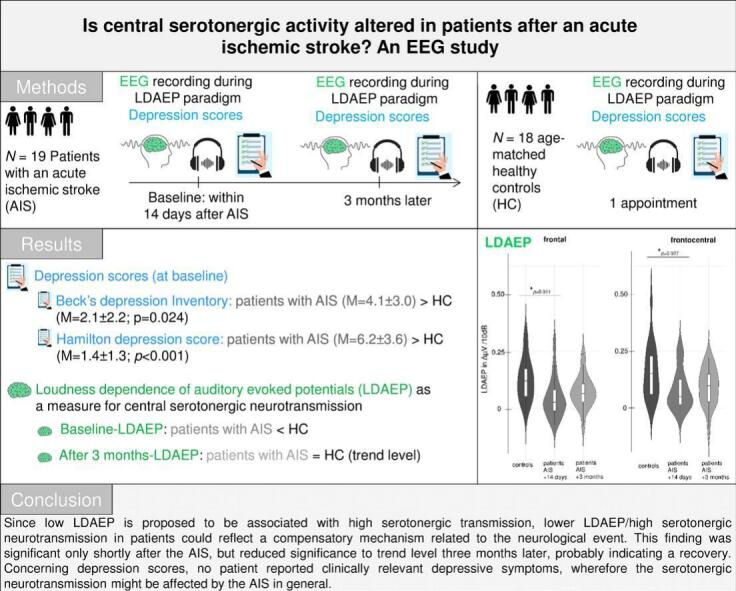

## 1 Introduction

Experiencing an acute ischemic stroke (AIS) is a very severe, life-threatening event and can lead to reduced abilities in daily life. Apart from neurological symptoms, AIS has been associated with the development of post-stroke depression in approximately 30 % of stroke patients (Liu et al., 2023, Robinson and Jorge, 2016). Suffering from post-stroke depression (PSD) further increases the rates of mortality (Bartoli et al., 2018).

As for major depressive disorder (Fakhoury, 2016), development of PSD is proposed to be based on dysfunctioning of serotonergic neurotransmission. Studies measuring serotonin in cerebrospinal fluid (CSF) found that lower levels of serotonin (5-HT) and its metabolite 5-hydroxyindoleacetic acid (5-HIAA) levels are associated with suicidal behavior in depression (Asberg, 1997, Hou et al., 2006). Similarly, PSD patients show reduced plasma and CSF serotonin and 5-HIAA compared to stroke patients without PSD (Bryer et al., 1992, Gao et al., 2008). Interestingly, the administration of selective serotonin reuptake inhibitors (SSRI), usually given to patients with major depressive disorder, to patients who experienced a stroke has been considered a preventive tool to avoid a depressive episode. A recent meta-analysis showed that early SSRI treatment was associated with a reduction in PSD occurrence when compared to patients receiving placebo, but also with relevant side effects (Richter et al., 2021). In addition to measuring serotonin levels in the cerebrospinal fluid, another research approach has focused on measuring central serotonergic activity using the loudness dependence of auditory evoked potentials (LDAEP), which is measured as the cortical response, assessed by electroencephalogram (EEG), to tones with different loudness levels. Here, a strong LDAEP, i.e. a strong reagibility, is proposed to be associated with reduced serotonergic transmission, and vice versa (Hegerl and Juckel, 1993) due to a negative association between the firing rate of the serotonergic neurons in the dorsal raphe nuclei (DRN) and sensory processing in the primary auditory cortex (Jacobs and Azmitia, 1992). The measurement of LDAEP is a non-invasive measurement and bears low risks in contrast to measuring CSF serotonin levels or observing SSRI effects. In patients with depression, LDAEP has been suggested to predict the responsiveness to serotonergic antidepressants (Gallinat et al., 2000, Juckel et al., 2007, Park et al., 2012). Findings concerning group differences between patients with depression and control groups have been inconsistent (Kangas et al., 2022). We found one work that describes higher LDAEP in 20 patients within 10 days after an acute stroke (and in a PSD subgroup) compared to healthy controls, which would suggest decreased central serotonergic neurotransmission (Rocco et al., 2007). Interestingly, patients with acute stroke but without PSD did not differ from the control group. A similar study reported higher LDAEPs in a PSD group (*n* = 7), who developed PSD 4 weeks after the event, compared to patients who did not develop depressive symptoms after a stroke (*n* = 30) (Meißner-Bendzko et al., 2024). There are no further studies assessing LDAEP in patients with stroke and PSD and a healthy control group. In addition, no study examined LDAEP and depression after a recovery period of several months. This is of particular interest since previous studies found even higher rates of PSD prevalence after 6 months (Fournier et al., 2020) and similar rates after 3 years (Schöttke et al., 2020) when compared to prevalence rates during the acute phase.

Gaining more knowledge on the development, progress and mechanisms of PSD, including changes in serotonergic functioning after a stroke, could help establish methods for preventing PSD in the future. Therefore, we compared LDAEP, as a non-invasive measure of central serotonergic activity, and depressive symptoms in patients who experienced an AIS, both shortly after the event and after three months, to participants who never experienced a cerebrovascular event. According to the previous work, we expected to find higher LDAEP and depression scores in the patient group compared to controls. We also expected to find higher LDAEP data in patients at 3 months after the event compared to the control group. The measurement of LDAEP could be easily integrated into clinical assessments in neurology departments and could provide important hints for potential PSD development.

## 2 Methods

The data presented here are part of a larger project examining biomarkers and post-stroke-depression in neurological patients treated on a specialized stroke unit in the neurology department of a university hospital (“prognostic markers of post-stroke depression”‘ short “PROMoSD” study; (Richter et al., 2022). The study was approved by the ethics committee of the Ruhr-University Bochum (No. 20-6862) and all procedures were in accordance with the Declaration of Helsinki of 1975.

### 2.1 Participants

This part of the project included 19 adult patients (>18 years) as well as 18 age-matched controls. Initially, 29 patients completed the first appointment but did not participate in the second testing session. Consequently, data from 19 patients are presented here. Patients had experienced an ischemic stroke attack within the past 14 days and were therefore treated as inpatients in the stroke unit of the neurology department; each diagnosis was confirmed by imaging (computer tomography or magnetic resonance imaging). All participants were required to have sufficient German language skills. Exclusion criteria were cognitive deficits, aphasia or other conditions which could interfere with psychiatric and neurological evaluation. For another part of this project including transcranial sonography, no transtemporal bone window was an additional exclusion criteria. Regarding the control group, participants were recruited from the community by public notices and had to be free of neurological or psychiatric conditions. None of them were excluded. They received detailed written study information and provided written informed consent.

### 2.2 Procedures

**Fig. 1:**
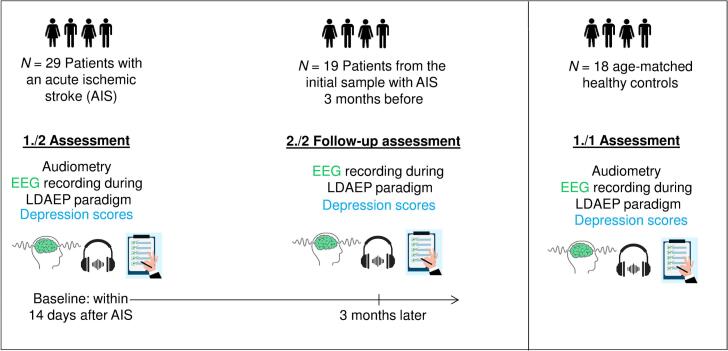
Graphical overview of study timeline including patient and control group sample sizes.

Shortly after the AIS, i.e. within 14 days of the AIS, audiometry, EEG recording and psychological testing (see below) were conducted with patients. Approximately three months after taking part in the first study appointment, patients were asked for another appointment for repeated EEG recording and psychometric assessments in order to evaluate changes over time. Control participants took part in a single study appointment with EEG-recording and psychological testing. See also Fig. 1 for a graphical representation of the study design.

### 2.3 Questionnaires

Before EEG recording, participants completed the Beck Depression Inventory (BDI-II; German Version (Kühner et al., 2007) and were assessed by a psychiatrist using the Hamilton Depression Scale (Hamilton, 1960) with 21 items. Sum scores were calculated for both questionnaires. For patients, the NIHSS score (National Institutes of Health stroke scale) was recorded, which ranges from 0 to 42, whereby higher scores indicate a greater stroke severity.

### 2.4 Audiometry

Prior to EEG-recording, sufficient hearing capacity of all participants were ensured by using an Oscilla USB 330 audiometer (medico A/S, Denmark). Hearing thresholds were determined for both ears for 25, 250, 500, 750, 1K, 1.5K, 2K, 3K, 4K, 6K, 8K Hz, respectively, whereas thresholds of at least 50 dB for 1000 Hz were required. For results see Supplemental material Table S1.

### 2.5 EEG-recording and analysis

The EEG was recorded using 32 passive non-polarizable Ag-AgCl electrodes including a ground and a reference electrodes (at FPz and FCz respectively) and an EOG electrode with a sampling rate of 250 Hz (online band-pass filter of 0.531–70 Hz). The EEG electrodes were mounted on an elastic cap (easy cap GmbH; Herrsching, Germany) according to the 10–20 system. We used the BrainAmp MR Amplifier and the BrainVision Recorder software (Version 1.20.001; Brain Products GmbH, Gilching, Germany). The impedances were kept at 10 kΏ or below during recording.

The auditory stimuli were presented binaurally using earphones (Sony Stereo Headphones MDR-1A, Sony® Corporation) and the Presentation ® software (Neurobehavioral Systems, Inc. Version 14.9; Berkeley, CA, https://www.neurobs.com). More detailed, sinus tones (1000 Hz, 40 ms, 10 ms r/f, ISI 1841–2239 ms, mean 2046 ms) of five different loudness levels (60, 70, 80, 90, 100 dB) were pseudorandomized presented to the participants. For each loudness level, 70 trials were presented, resulting in 350 total trials.

BrainVision Analyzer 2.2 (Version 2.2.1.8266; Brain Products GmbH, Gilching, Germany) was used for data analysis. EEG Data has been re-referenced to the mastoid electrodes and filters were applied (notch-filter for 50 Hz; high-and low pass filters 0.5 and –30 Hz). The EOG electrode was used for correction of eye movements by ICA. The recordings were segmented according to the five loudness levels in epochs of 800 ms length (-200 ms before and 600 ms after stimulus onset). Baseline correction was applied for the -200 ms before stimulus onset. EEG data showing voltage above or below 100 μV were marked as bad sections and excluded during later segment averaging. The remaining trials were averaged for each loudness level, whereby the first 5 trials of each of the 5 loudness levels were not included in the analysis. At least 25 artifact-free trials were required for each loudness level for each dataset to be included into further statistical analyses. For 60 dB 58.6 trials (*SD* = 7.7) were included, for 70 dB 58.7 trials (*SD* = 8.0), for 80 dB 58.3 trials (*SD* = 8.8), for 90 dB 58.8 trials (*SD* = 8.2) and for 100 dB 57.4 trials (*SD* = 9.8) remained after artifact rejection. These trials were averaged. Averages for the N1 and P2 peak amplitudes in µV were exported for each loudness level. Here, the N1-component has been determined as the first negative peak after stimulus presentation (between 70 and 180 ms) and the P2-component was identified as the second positive peak between 150 ms and 280 ms. Differences between N1-peak and P2-peaks for all electrodes were calculated for each loudness level. For the LDAEP, the slopes belonging to tangents fitted for all combinations of association pairs of loudness levels (e.g. loudness level and N1-P2 difference of 100 dB and 60 dB) were calculated. Finally, the median for the resulting 10 slopes has been calculated and used for further statistical analysis. Median slopes were calculated for electrodes F3, F4, F7, F8, Fz, FC1, FC2, FC5, FC6, C3, C4, Cz, CP1, CP2, CP5, CP6. We calculated averages for frontal electrodes including F3, F4, F7, F8 and Fz electrodes. The frontocentral LDAEP average contained FC1, FC2, FC5, FC6 electrodes and the central LDAEP constitutes of C3, C4, Cz electrodes. Finally, centroparietal LDAEP was calculated based on CP1, CP2, CP5 and CP6.

### 2.6 Statistical analyses

Depression scores (BDI and Hamilton) were compared between patients and controls by using Mann-Whitney-*U-*tests and between timepoints by Wilcoxon-tests. As there was homogeneity of the error variances (assessed by Levene’s test; *p* > 0.05) and homogeneity of covariances (assessed by Box’s test; *p* > 0.05), we calculated a mixed-model ANOVA with the factors group (patients vs. controls) and position (frontal, frontocentral, central, centroparietal). For the comparison of shortly after admission vs. three months LDAEP within the patients group, an ANOVA was computed with the factors timepoint (14 days vs. 3 months) and position (frontal, frontocentral, central, centroparietal). Finally, we compared the LDAEP of controls with the LDAEP of patients at the three months appointment by using a mixed model ANOVA with factors group (patients vs. controls) and position (frontal, frontocentral, central, centroparietal). Greenhouse-Geisser corrected values are reported and posthoc testing was conducted by using dependent and independent *t*-tests (two-tailed). Partial *η^2^* and Cohen’s *d* are provided as measures of effect sizes and the statistical power reached for our given sample sizes and the resulting effect sizes are calculated by using G*Power 3.1.9.2. Control analysis for the hearing capacity comprise an ANOVA with factors (patients group vs. control group), ear (left, right) and frequency (125 Hz, 250 Hz, 500 Hz, 750 Hz, 1K Hz, 1.5K Hz, 2K Hz, 3K Hz, 4K Hz, 6K Hz, 8K Hz). To investigate whether LDAEP is specifically altered over the stroke region, we calculated Spearman correlations between LDAEP at the first timepoint in patients with AIS over the affected left/right/both hemispheres and the demarcation size. Subsequently, a more refined correlation analysis was conducted, focusing on frontal and parietal regions, along with the demarcation size. Due to the nature of electroencephalography, its capacity is inherently constrained to superficial brain regions. Consequently, correlations could not be computed for deeper brain regions affected by stroke, such as the thalamus, pons, and cerebellum.

## 3 Results

The results of the present study contain findings of group and stroke characteristics (3.1), as well as findings from LDAEP assessments shortly after the AIS when compared to the healthy control group (3.2). In addition, we present comparisons between patients at 14 days versus 3 months (3.3) and comparisons between control participants and patients re-assessed after three months (3.4). Control analyses include the investigation of the impact of hearing ability on LDAEP (3.5) and the association between AIS affected region and LDAEP (3.6).

### 3.1 Psychometric group characteristics

The age of both groups did not differ significantly (age: Patient group *M* = 64.6, *SD* = 11.8, control group *M* = 64.0, *SD* = 5.7). Neurological patients showed higher scores in BDI (*M* = 4.1, *SD* = 3.0) and Hamilton (*M* = 6.2, *SD* = 3.6) shortly after the AIS than control participants (BDI *M* = 2.1, *SD* = 2.2; HAMD *M* = 1.4, *SD* = 1.3). However, the BDI range was 0–12 in patients (controls 0-8), which is below the cutoff of 13 that would indicate a mild depression. For the Hamilton Scale, the range was 2–15 in patients (controls 0-4), whereas the cutoff of 8 was reached by five patients. However, since these scores are below 17, which is considered a clinically relevant score for a moderate depression, both groups are considered as not being depressive. Three months after the event, the BDI range was 0–13 (*M* = 5.7, *SD* = 3.8) and for Hamilton it was 0-13 (*M* = 5.3, *SD* = 3.4), which suggests no development of a depressive episode (BDI 14 days vs. 3 months: *Z* = -1.926, *p* = 0.054; HAMD 14 days vs. 3 months: *Z* = -1.49, *p* = 0.136). Briefly after the neurological event, the NIHSS was on average 1.7 (*SD* = 1.32; range 0-5). For details and characteristics of the AIS, see Table 1.-

**Table 1:** Demographic and clinical characteristics of patients (within 14 days after the AIS) and control participants.

-	**Patient group**	**Control group**	**Statistics**
Age (mean (SD))^a^	64.6 (11.8)	64.0 (5.7)	*t*(26.4) = − 0.191, *p* = 0.850, *d* = − 0.062)
Gender (female/male)^b^	9/9	5/14	χ^2^(1) = 2.20, *p* = 0.138
Marital status (single/divorced/married/widowed)^b^	2/2/11/3	2/3/13/0	χ^2^(3) = 3.37, *p* = 0.338
BDI^c^	4.1 (3.0)	2.1 (2.2)	*U* = 97.0, *Z* = − 2.29, *p* = 0.024
HAMD^c^	6.2 (3.6)	1.4 (1.3)	*U* = 27.0, *Z* = − 4.42, *p* < 0.001
NIHSS	1.7 (1.33)		
AIS characteristics (patients)*			
Hemisphere demarcation (right/left/both n (%)			5 (26.3 %)/13 (68.4 %)/1 (5.3 %)
Region demarcation (frontal/parietal/thalamus/cerebellum/pons/ multiple n (%)			4 (21.2 %)/3 (15.8 %)/3 (15.8 %)/4 (21.2 %)/2 (10.5 %)/3 (15.8 %)
Mean (total) size of demarcation (SD) in cm^3^*			8.3 (4.0)

BDI = Beck’s depression Inventory; HAMD = Hamilton depression score; NIHSS = National Institutes of Health stroke scale.

a comparison of variables by *t*-test; mean and SD reported.

b comparison of variables by chi-square test; frequencies reported.

c comparison of variables by Mann-Whitney-*U* test; mean and SD reported.

* n = 19 for region and hemisphere of demarcation, n = 18 for size.

### 3.2 Comparison of LDAEP between patients (shortly after AIS) and controls

The ANOVA showed a significant main effect of group (*F*(1,35) = 4.35, *p* = 0.044, partial *η^2^* = 0.111), a main effect of position (*F*(1.79,62.71) = 10.01, *p* < 0.001, partial *η^2^* = 0.222) and an interaction of position with group (*F*(1.79) = 3.53, *p* = 0.040, partial *η^2^* = 0.092). A post hoc test for the main effect of group showed lower LDAEP in patients compared to control participants averaged across all electrodes (LDAEP of all electrodes: patient group: *M* = 0.072, *SD* = 0.077; control group: *M* = 0.133, *SD* = 0.095; *t*(35) = 2.17, *p* = 0.037, *d* = 0.712; power = 0.56).

**Fig. 2:**
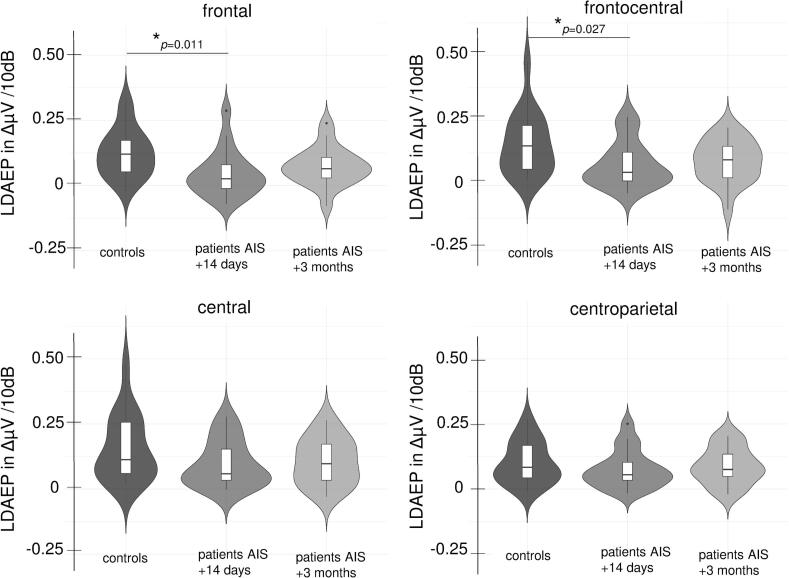
Graphical presentation of LDAEP (violin plots with superimposed averages) in controls and patients briefly after the AIS and after 3 months for frontal, frontocentral, central and centroparietal electrodes.

**Fig. 3:**
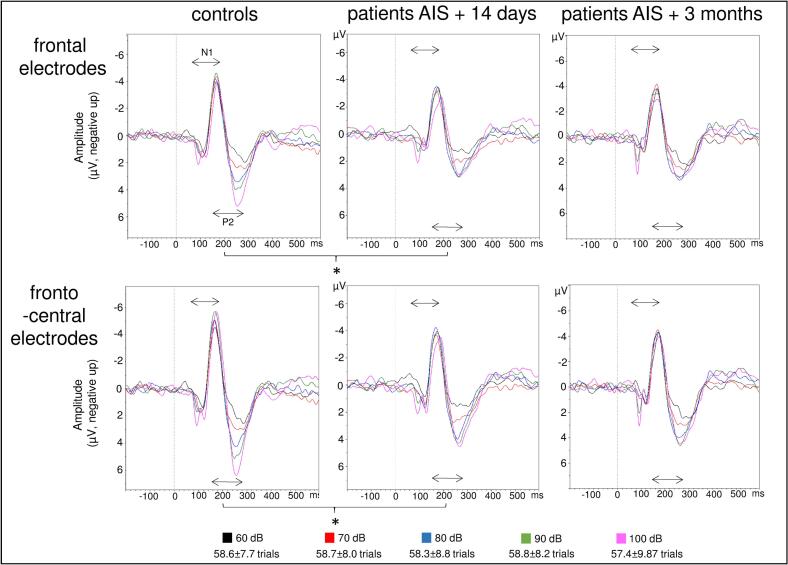
LDAEP waveforms for controls and patients at 0–14 days and three months after an AIS over frontal (F3, F4, F7, F8, Fz averaged) and frontocentral (FC1, FC2, FC5, FC6 averaged) electrodes. Significant differences are marked by *; N1 and P2 time windows are indicated by double arrows.

The interaction of position x group showed significantly lower LDAEP in patients compared to controls over frontal and frontocentral electrodes (frontal: patient group *M* = 0.045, *SD* = 0.089 vs. control group *M* = 0.125, *SD* = 0.092, *t*(35) = 2.68, *p* = 0.011, *d* = 0.880, power = 0.74; frontocentral: patient group *M* = 0.081, *SD* = 0.086 vs. control group *M* = 0.158, SD = 0.116, *t*(35) = 2.31, *p* = 0.027, *d* = 0.75, power = 0.60), whereas no differences were observed over central and centroparietal electrodes (central: patient group *M* = 0.097, *SD* = 0.088 vs. control group *M* = 0.156, *SD* = 0.125, *t*(35) = 1.67, *p* = 0.103, *d* = 0.550; centroparietal: patient group *M* = 0.076, *SD* = 0.072 vs. control group *M* = 0.101, *SD* = 0.080, *t*(35) = 1.01, *p* = 0.312, *d* = 0.331, see Fig. 2, Fig. 3). When applying Bonferroni correction for multiple post hoc testing, the differences for frontal electrodes would remain significant (4 comparisons: *p* < 0.013).

The main effect of position indicated that LDAEP was lowest over frontal and centroparietal electrodes and higher over frontocentral and central electrodes (frontal *M* = 0.084, *SD* = 0.098 vs. frontocentral *M* = 0.119, *SD* = 0.108: *t*(36) = -4.13, *p* < 0.001, *d* = -0.679; frontal *M* = 0.084, *SD* = 0.098 vs. central *M* = 0.126, *SD* = 0.110: *t*(36) = -3.20, *p* = 0.003, *d* = -0.526; frontocentral *M* = 0.119, *SD* = 0.108 vs. centroparietal *M* = 0.088, *SD* = 0.076: *t*(36) = 3.18, *p* = 0.002, *d* = 0.523; central *M* = 0.126, *SD* = 0.110 vs. centroparietal *M* = 0.088, *SD* = 0.076: *t*(36) = 4.63, *p* < 0.001, *d* = 0.761; frontal vs. centroparietal and frontocentral vs. central were not significant).

### 3.3 Comparisons within patient group: LDAEP shortly after AIS vs. Three months

The ANOVA with factors timepoint (14 days/three months) and position (frontal, frontocentral, central, centroparietal) showed no significant main effect, nor interactions of timepoint. Again, the main effect of position was observed (*F*(1.83,32.83) = 4.70, *p* = 0.018, partial *η^2^* = 0.207).

However, when computing an exploratory *t*-test between 14 days and three months data for frontal electrodes, a tendency towards higher LDAEP values at the three-months measurement emerged (14 days: *M* = 0.045, *SD* = 0.089 vs. three months: *M* = 0.073, *SD* = 0.073, *t*(18) = -1.68, *p* = 0.110, *d* = -0.386; see also Fig. 2).

### 3.4 Comparison of LDAEP between patients (after three months) and controls

The ANOVA with factors group (patient group vs. control group) and position (frontal, frontocentral, central, centroparietal) showed only a trend for the main effect for group (*F*(1,35) = 2.57, *p* = 0.118, partial *η^2^* = 0.068) and for the interaction of position with group (*F*(1.92) = 2.55, *p* = 0.088, partial *η^2^* = 0.068). There was also a main effect of position (*F*(1.92,67.03) = 7.37, *p* = 0.001, partial *η^2^* = 0.174). Thus, the differences between groups observed shortly after the neurological event were not observed when LDAEP was measured three months later.

### 3.5 Control analysis

In order to check whether hearing ability might have confounded the data, we calculated an ANOVA with factors group (patient group vs. control group), ear (left, right) and frequency for audiometry thresholds. Apart from the main effect of frequency (*F*(3.10,108.62) = 68.592, *p* < 0.001, partial *η^2^* = 0.662), no other main effect or interaction appeared, indicating that there was no difference in hearing ability between groups.

### 3.6 Correlations between LDAEP and characteristics of the AIS

The correlation between LDAEP over the hemisphere affected by AIS (left, right or both) and the demarcation size was not significant (*r* = -0.063, *p* = 0.804, *n* = 18). The correlation within the patient group with frontal and parietal brain regions affected was also not significant (*r* = 0.257, *p* = 0.623, *n* = 6).

## 4 Discussion

The present study aimed to investigate whether central serotonergic system functioning would be altered after an AIS and whether it is related to the development of a so-called post-stroke depression briefly after the event and after three months. In our sample, no patient fulfilled the clinical criteria for a depressive episode, therefore, we cannot draw conclusions regarding post-stroke depression. Thus, the main question remains whether an AIS and its recovery affect serotonergic neurotransmission. In contrast to our hypothesis, patients showed significantly lower LDAEP compared to control participants especially over frontal electrodes briefly after the event. This difference only reached trend level when LDAEP of controls was compared to LDAEP of patients at three-months after the event. In addition, there was a tendency towards higher LDAEP values at the three-month measurement compared to the 14-day measurement. These findings indicate that LDAEP was lowest briefly after the AIS and “recovers” after a few months and finally does not differ from controls’ LDAEP. Thus, the AIS was related to lower LDAEP, which would suggest increased serotonergic neurotransmission. In previous studies focusing on LDAEP in post-stroke patients, there was no difference between stroke patients without depression and controls (Rocco et al., 2007), but between PSD patients and controls (Rocco et al., 2007) and between PSD patients and stroke patients without depressive symptoms (Meißner-Bendzko et al., 2024). As we developed our hypothesis based on prior data, we anticipated a lower level of serotonergic activity. However, given the focus of the previous study on patients with PSD, direct comparisons between the two groups, comprising AIS patients with and without PSD, are not feasible. Therefore, it can be posited that the mechanism in these disparate groups may vary, constituting a salient objective for subsequent research endeavors. To the best of our knowledge, there is no other LDAEP study on serotonergic neurotransmission after acute ischemic cerebrovascular events. However, there are hints for enhanced serotonin activity in the hippocampus, which exerts protection against neuronal damage after ischemia in animals (Abdel-hameed et al., 2021). In humans, one study reported lower serum serotonin levels in post-stroke patients after a 6-week rehabilitation when compared to blood samples withdrawn before rehabilitation (Siotto et al., 2021). Serotonin levels in the plasma were found to be correlated to CSF serotonin levels and levels in both samples were lower in a PSD group than in patients with stroke without depression 15 and 30 days after the stroke (Gao et al., 2008). Further research suggests altered monoamine metabolism and altered availability of serotonin precursors in the brain after strokes (Aquilani et al., 2004, Ormstad et al., 2013, Tang et al., 1989). How this affects neurotransmission in the brain has not been examined before. Data of another part of this project focusing on a patient group showed that a hypoechogenic brainstem raphe, assessed by transcranial sonography, is a strong and independent predictor of PSD at three months after a stroke (Richter et al., 2024). Since reduced echogenic brainstem raphe signal has been related to dysfunctions of the serotonergic system and depression, this finding supports the idea of altered serotonergic functioning in patients with PSD (Krogias and Walter, 2016). Finally, research on SSRI reported a reduction in PSD occurrence when SSRIs were administered (Gu et al., 2018, Richter et al., 2021), as well as a reduction in defective neurologic, motor and cognitive functioning under SSRI treatment in non-depressed patients after stroke (Gu et al., 2018, Kalbouneh et al., 2022, Su et al., 2021). Taken together, there are hints for altered serotonergic functioning after AIS and for an association with PSD development.

The present study bears several limitations: First, we cannot draw conclusions regarding PSD, because the patients included did not develop a PSD, therefore larger studies including patients with PSD should be conducted. Second, the sample size is relatively small, so a further study aiming to replicate the findings is important. Third, because the severity of neurological symptoms was low in the present sample (NIHSS mean = 1.7), the results might not be generalizable to more severely affected patients. Forth, as the gender distribution was not balanced in the control group, LDAEP might have been affected, since gender is proposed to impact LDAEP strength (Min et al., 2012).

In our sample, no patient reported clinically relevant depressive symptoms, wherefore the serotonergic neurotransmission might be affected by the AIS in general, and in a different way in patients developing PSD. Thus, the lower LDAEP, i.e. higher serotonergic neurotransmission briefly after the event might reflect a compensatory strategy of the body to avoid PSD. Indeed, a considerable body of research has posited that SSRIs can trigger advantageous processes, thereby mitigating the incidence of brain injury and functional impairment subsequent to AIS. The proposed mechanisms encompass enhanced neuroplasticity, neuroprotection, augmented cerebral blood flow autoregulation, and adaptation of the adrenergic neurohormonal system (Siepmann et al., 2015). However, whether the bodies of individuals who have experienced AIS increase serotonin availability in order to induce endogenous ‘repair mechanisms’ conveyed by serotonin remains unclear. After three months, the patients did not develop a PSD and LDAEP values increased and were no longer different from controls. Since the idea of a „compensatory strategy” is highly speculative, future research is necessary to further investigate the mechanisms and processes in those patients developing PSD. In addition, it is not known whether these effects would also be observable under acute stress or inflammation. As an alternative explanation, the altered LDAEP could be simply related to brain lesions caused by the AIS. Such lesions would lead to altered neuronal processing in general, which would not selectively be related to serotonergic neurotransmission. By assessing correlations between LDAEP over the affected brain regions and lesion size, we aimed to address this issue. As correlations were not significant, this theory has not been confirmed. However, given the limited sample sizes for the regions that are detectable by electroencephalography (EEG), it is not possible to draw definitive conclusions based on the data obtained. Future research would be required to focus on the specific LDAEP changes over brain regions affected by AIS.

### 4.1 Conclusion

The present exploratory study aiming to assess central serotonergic functioning in patients with AIS, revealed lower LDAEP, which would indicate higher serotonergic neurotransmission, in patients compared to a control group. This finding was significant only shortly after the AIS, but was reduced to trend level three months later. Speculatively, the LDAEP decrease could reflect a compensatory mechanism related to the neurological event. In summary, the present study shows that experiencing an AIS led to short-term alterations in LDAEP. As these findings need to be considered preliminary, future research should aim to further investigate the relationship between AIS, central serotonergic neurotransmission, and the development of PSD, as well as the role of AIS symptom severity. It is conceivable that LDAEP measurements briefly after AIS could assist in identifying patients who would benefit from SSRI treatment to reduce functional impairments and PSD.

## Declaration of Competing Interest

The authors declare that they have no known competing financial interests or personal relationships that could have appeared to influence the work reported in this paper.
