# Supplementary material for: Increased central serotonergic activity in patients after an acute ischemic stroke. An EEG study
Source: Clin Neurophysiol Pract. 2025 Oct 17;10:480–6. doi: 10.1016/j.cnp.2025.10.003 (PMC12569835; doi:10.1016/j.cnp.2025.10.003)
Supplement: Supplementary Data 1 [file mmc1.docx]

Supplemental Material

## Extended results

### Audiometry

**Table S1:** Audiometry thresholds (in dB) in patients (within 14 days after the AIS) and control participants (mean and SD).

|  | **Left ear** | | **Right ear** | |
| --- | --- | --- | --- | --- |
| **Frequency** | **Patients group** | **Control group** | **Patients group** | **Control group** |
| 125 Hz | 24.7 9.5 | 23.3 6.6 | 23.7 11.3 | 22.2 7.3 |
| 250 Hz | 20.0 6.5 | 16.7 8.6 | 22.9 6.3 | 20.8 10.0 |
| 500 Hz | 19.7 5.9 | 15.3 7.9 | 22.4 8.4 | 17.2 9.4 |
| 750 Hz | 21.1 8.6 | 17.5 7.9 | 21.6 8.7 | 18.9 6.3 |
| 1K Hz | 20.0 9.6 | 18.3 8.6 | 21.8 10.8 | 17.8 6.2 |
| 1.5K Hz | 22.6 9.6 | 20.3 13.0 | 25.3 13.3 | 20.8 9.1 |
| 2K Hz | 27.9 12.8 | 25.3 12.7 | 30.0 12.8 | 24.9 9.7 |
| 3K Hz | 34.2 15.7 | 30.3 15.8 | 36.1 13.9 | 27.8 10.7 |
| 4K Hz | 43.7 18.1 | 41.9 20.0 | 44.7 16.0 | 37.2 16.4 |
| 6K Hz | 46.8 18.9 | 42.5 23.3 | 47.4 16.3 | 40.8 18.7 |
| 8K Hz* | 53.6 16.6 | 45.3 21.7 | 59.1 18.2 | 41.4 21.3 |

* *Missing variables were replaced by means of the respective groups (8K left n=1 in controls and n=8 in patients; 8K right n=7 in patients).*
